# Changing paradigm of antibiotic resistance amongst *Escherichia coli* isolates in Indian pediatric population

**DOI:** 10.1371/journal.pone.0213850

**Published:** 2019-04-17

**Authors:** Taru Singh, Praveen Kumar Singh, Sajad Ahmad Dar, Shafiul Haque, Naseem Akhter, Shukla Das

**Affiliations:** 1 Department of Microbiology, University College of Medical Sciences & GTB Hospital (University of Delhi), Delhi, India; 2 Research and Scientific Studies Unit, College of Nursing and Allied Health Sciences, Jazan University, Jazan, Saudi Arabia; 3 Department of Laboratory Medicine, Faculty of Applied Medical Sciences, Albaha University, Albaha, Saudi Arabia; Tallinn University of Technology, ESTONIA

## Abstract

Antimicrobial resistance happens when microorganisms mutates in manners that render the drugs like antibacterial, antiviral, antiparasitic and antifungal, ineffective. The normal mutation process is encouraged by the improper use of antibiotics. Mutations leading to quinolone resistance occur in a highly conserved region of the quinolone resistance-determining region (QRDR) of DNA *gyrA*se and topoisomerase IV gene. We analyzed antibiotic resistant genes and single nucleotide polymorphism (SNP) in *gyrA* and *parC* genes in QRDR in 120 *E*. *coli* isolates (both diarrheagenic and non-pathogenic) recovered from fresh stool samples collected from children aged less than 5 years from Delhi, India. Antibiotic susceptibility testing was performed according to standard clinical and laboratory standards institute (CLSI) guidelines. Phylogenetic analysis showed the clonal diversity and phylogenetic relationships among the *E*. *coli* isolates. The SNP analysis depicted mutations in *gyrA* and *parC* genes in QRDR. The sul1 gene, responsible for sulfonamide resistance, was present in almost half (47.5%) of the isolates across the diseased and healthy samples. The presence of antibiotic resistance genes in *E*. *coli* isolates from healthy children indicate the development, dissemination and carriage of antibiotic resistance in their gut. Our observations suggest the implementation of active surveillance and stewardship programs to promote appropriate antibiotic use and minimizing further danger.

## Introduction

Childhood diarrhoea, a major cause of child mortality globally, affects an estimated 2.2 million children in developing countries alone [1]. Antimicrobial drugs have played an important role in reducing death toll caused by infectious diseases. However, infections caused by multidrug-resistant (MDR) organisms have emerged as a huge threat to the community and hospitalized patients. In this regard the emergence of MDR *E*. *coli* isolates from human, animal and environmental sources have posed a major concern worldwide [2, 3].

Study of antimicrobial resistance and regional variation is vital for the development and implementation of interventional strategies. *E*. *coli*, though a commensal, has acquired resistance to various groups of antimicrobials at a rapid rate in diverse geographical areas, emphasizing the need of antimicrobial resistance (AMR) surveillance, especially in low resource settings where the cost of patient management escalates once hospitalized and colonized by drug resistant microbes. Early studies suggest that *E*. *coli* isolates recovered from the stools of healthy children have significantly higher rates of multi-drug resistances in China in comparison to the developed countries [4]. Similar observation in rest of Asia and South America, thus accentuate the threat of widespread drug resistance and the urgent need of implementation of future preventive measures and planning strict policy of antibiotic usage [5]. Moreover, co-evolution of virulence factors with antibiotic resistance genes have eventually contributed to the adaptive potential of these resistant microbes and long-term survival [6, 7].

Interestingly, existence of antibiotic resistance has started from the era of penicillin discovery, prompting the start of a national surveillance program namely ‘Resistance Map’ (www.resistancemap.org) demonstrating the occurrence of resistance two decades back involving India and China as the main contributors [8–10]; {Figure A to D in S1 Fig}. In India, very high resistance was reported against ampicillin and nalidixic acid along with an increased resistance to third generation cephalosporins, fluoroquinolone and carbapenems in *E*. *coli* [11].

Mobile genetic elements like integrons contain many antibiotic resistance determinants in *E*. *coli*. Integrons can also be defined as the systems for site-specific recombination found in transposons, plasmids and chromosomes [12]. Gene cassettes with multiple antibiotic resistance genes in the form of clusters can be found in integrons which contribute majorly to the development of multiple antibiotic resistances [13].

The rapid spread of β-lactamases resistance, led by mobile genetic elements, amongst susceptible bacteria and acquisition of plasmid-mediated β-lactamases such as extended-spectrum β-lactamases–ESBL (*TEM*, *SHV*, *CTX-M* and *OXA*), and class C plasmid-mediated AmpC β-lactamases–ABL (*ACT*, *CMY* and *DHA*) amongst *E*. *coli* are well documented [14–16]. Metallo-β-lactamases—MBL (*VIM*, *IMP* and the recent *NDM*), have further led to limitations in the treatment options [17]. ESBL producers are no longer associated with hospital infections only rather community acquired isolates are now adding to burden of drug resistance [18]. Phenotypic methods have poor detection performance; consequently rampant misidentification of the drug resistant genes have led to the current disastrous therapeutic failure in life threatening infections [19, 20].

Genetic elements involving *sul1*, *sul2 and sul3* genes [21–26] and *tetA* (A), *tetB* (B), *tetC* (A), *tetD* (A), *tetE* (A) and *tetG* (A) genes are other important targets conferring resistance to Sulphonamides and Tetracyclines respectively [27, 28]; and mutations in the quinolone resistance-determining region (QRDR) of *gyrA* or *gyrB* subunits of DNA *gyrA*se and *parC* genes or *parE* subunits of topoisomerase IV for fluoroquinolones resistance have also been described in several infections leading to treatment collapse [29–39]. Further, alterations in drug targets causing decreased cellular accumulation of quinolones and accompanied major multidrug efflux pump, *AcrAB*, may be contributing further to fluoroquinolone resistance [40–46].

The *E*. *coli* populations categorized into eight major phylogenetic groups namely A, B1, B2, C, D, E, F (belonging to *E*. *coli sensustricto)* and clade I (belonging to *Escherichia* clade) [47] have a vast genetic substructure within the species.

In view of the recent progression of antibiotic resistance in children under five, not only in clinical but also in community settings; we carried out this study to find the prevalence of different antibiotic resistance genes, to analyze point mutation in QRDR of fluoroquinolones and to detect the distribution of these resistance genes in different phylogroups.

## Materials and methods

### Study design

During the study period (July 2013 to July 2015), a total of 120 stool samples were collected from children up to five years of age. Each group included 40 subjects and were categorized as diarrhoeal (O), non-diarrhoeal (I) and healthy (C). The subjects were provided with relevant information about the study and were included with written informed consent from their parents/guardians. The study was approved by the Institutional Ethics Committee for Human Research (IEC-HR) of the University College of Medical Sciences (University of Delhi), Delhi and was carried out in accordance with its recommendations.

### Sample collection and processing

Conventional biochemical tests were used to identify the recovered *E*. *coli* [48] before performing the PCR for the 16SrRNA gene, which was also used as an internal quality control [49]. Antibiotic resistance was determined by the agar diffusion method (Kirby-Bauer method) using 16 antibiotics (HiMedia Laboratories, Mumbai, India) under four different classes namely aminoglycosides, fluoroquinolones, β-lactams and quinolones. The *E*. *coli* isolates were classified as sensitive or resistant according to CLSI guidelines at 24 hours of incubation at 37°C [48, 50, 51].

### DNA extraction, primers and analysis

DNA was extracted using the commercial kit (Real Biotech Corporation, Taiwan) and conventional PCR was performed for identification of genes associated with antibiotic resistance, single nucleotide polymorphism (SNP) and phylogenetic groups. Primers used are described in Table 1 [22,35,36,49,52–58].

**Table 1:** Details of the primers used.

Multiplex PCR	Gene	Primer sequence (5’–3’)	PCR product (bp)	Annealing temperature (°C)	Reference
**Aminoglycoside adenylyl transferases**	*AadA (aadA1 or aadA2)*	GCTCTTCAGCAATATCACGG GCAGCGCAATGACATTCTTG	282	60	[52]
SNP Detection					
**DNA gyrase**	*GyrA*	CTCCTCCCAGACCAAAGACA TCACGACCGATACCACAGCC-	447	60	[35, 36]
**DNA topoisomerase IV**	*ParC*	AAACCTGTTCAGCGCCGCATT GTGGTGCCGTTAAGCAAA	395	54	[35, 36]
Antibiotic Resistance Genes					
**Tetracycline**	*TetA-F* *TetA-R*	GTAATTCTGAGCACTGTCGC CTGCCTGGACAACATTGCTT	937	62	[53]
**Sulphonamides**	*Sul1-F* *Sul2-R*	TGGTGACGGTGTTCGGCATTC GCGAGGGTTTCCGAGAAGGTG	789	63	[22]
**Gentamicin**	*AacC1-F* *AacC1-R*	ACCTACTCCCAACATCAGCC ATATAGATCTCACTACGCGC	169	60	[54]
**Tetracycline**	*TetB-F* *TetB-R*	CTCAGTATTCCAAGCCTTTG CTAAGCACTTGTCTCCTGTT	416		[54]
**Tetracycline**	*TetC-F* *TetC-R*	TCTAACAATGCGCTCATCGT GGTTGAAGGCTCTCAAGGGC	570		[54]
**ESBL**	*TEM*	AGTGCTGCCATAACCATGAGG CTGACTCCCCGTCGTGTAGATA	431		[55]
	*SHV*	GATGAACGCTTTCCCATGATG CGCTGTTATCGCTCATGGTAA	214		
	*OXA*	ATTATCTACAGCAGCGCCAGTG TGCATCCACGTCTTTGGTG	296		
	*CTX-M*	GACAAAGAGAGTGCAACGGATG TCAGTGCGATCCAGACGAAA	501		
**MBL**	*blaNDM-1*	ATTAGCCGCTGCATTGAT CATGTCGAGATAGGAAGTG	154	55	[56]
	*blaIMP*	TTGACACTCCATTTACAG GATTGAGAATTAAGCCACTCT	139		[57]
	*blaVIM*	GATGGTGTTTGGTCGCATA CGAATGCGCAGCACCAG	390		
**ABL**	*CMY*	GCTGCTCAAGGAGCACAGGAT CACATTGACATAGGTGTGGTGC	520	60	[58]
	*DHA*	AACTTTCACAGGTGTGCTGGGT CCGTACGCATACTGGCTTTGC	405		
	*ACT-1*	TCGGTAAAG CCGATGTTG CGG CTT CCA CTG CGG CTG CCA GTT	302		
**Reference gene**	*16SrRNA*	CCCCCTGGACGAAGACTGAC ACCGCTGGCAACAAAGGATA	401		[49]

All the isolates were screened for phylogenetic groups A, B1, B2, C, D, E, F and Clade I using quadruplex multiplex PCR as described by Clermont et al. [47]. The presence of *chuA* gene represents groups B2 and D and absence represents groups A and B1. Group B2 and group D are being differentiated by *yjaA* gene.

Sequences of PCR product analyzed, commercially by Helix Biosciences (Bangalore, India), were matched with nucleotide sequences available at GenBank using the BLAST program to identify the most similar sequences [59]. Few of the sequences identified from the current study were submitted to the GenBank database and accession numbers obtained. Multiple alignments of sequenced nucleotides were carried out using Clustal W2 (version 2.0.10). Neighbor-joining method was used to construct tree in MEGA 6.0 [60, 61].

### Statistical analysis

Statistical analysis was done using Sigma Stat Statistics Software (SPSS) package. The Chi-square test and Fisher’s exact test were used to determine the statistical significance of data. The *p*-value < 0.05 was considered significant.

## Results

### Isolation of antibiotic resistant genes and SNP

**Fig 1:**
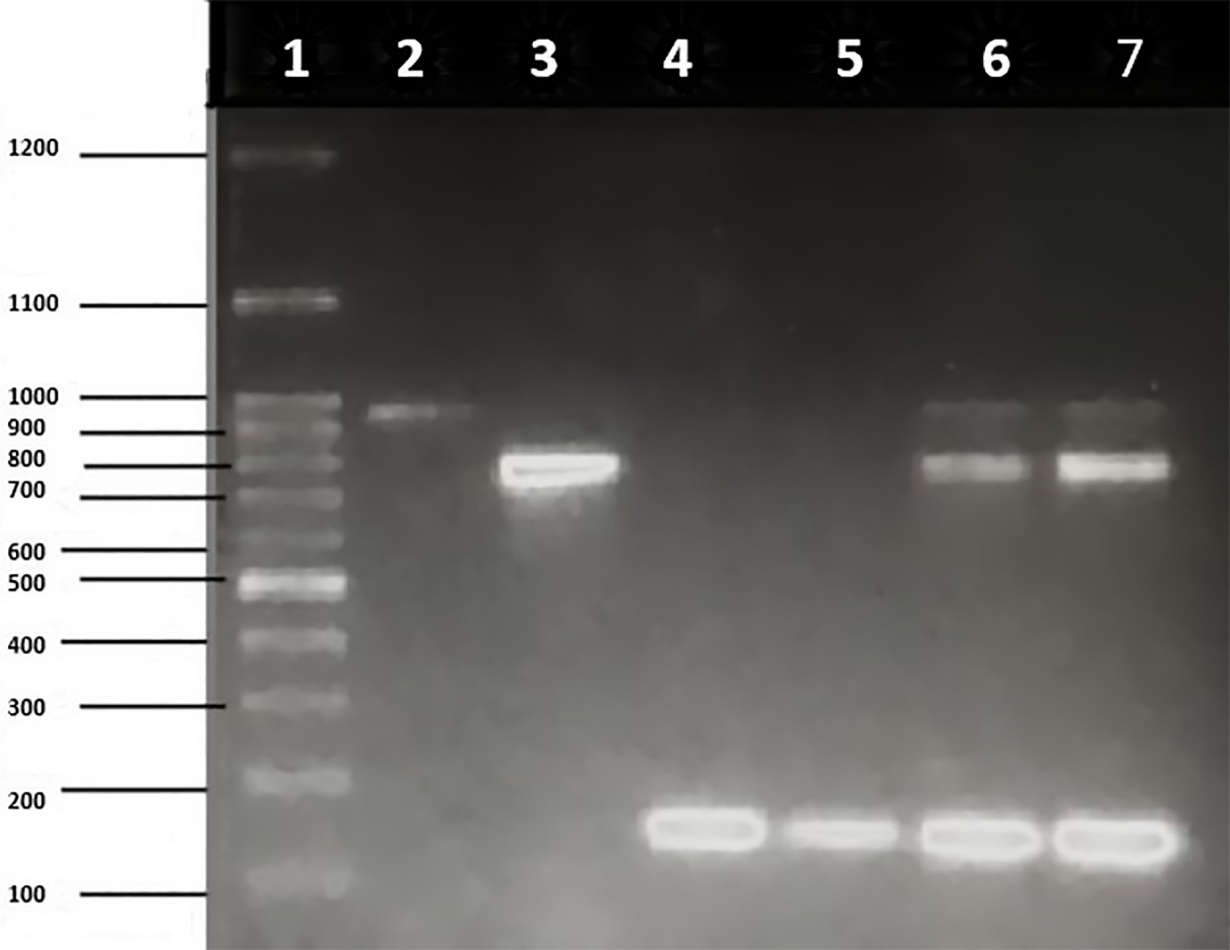
Multiplex PCR for antibiotic resistance genes (*tetA*, *sul1* and *AacC1*) on 1.5% agarose gel. Lane 1: molecular weight marker (100 bp), lane 2: *tetA* (937 bp), lane 3: *sul1* (789 bp), lane 4–5: *AacC1* (169 bp), lane 6–7: *tetA + sul1 + AacC1*.

**Fig 2:**
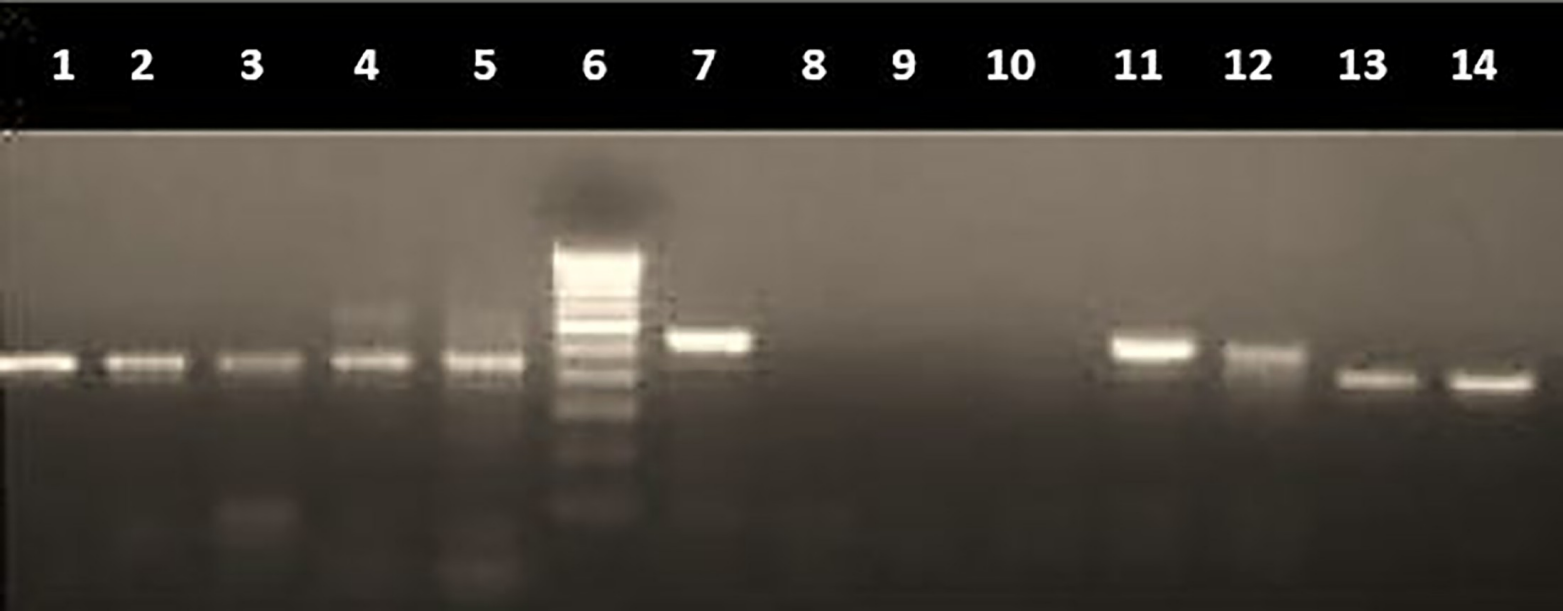
PCR for antibiotic resistance genes (*tetB* and *tetC*) on 1.5% agarose gel. Lane 1–3: *tetB* (416 bp), lane 4–5: *tetB + tetC* (570 bp), lane 6: molecular weight marker (100 bp), lane 7: *tetC*, lane 8–10: negative isolates, lane 11–12: *tetC* and lane 13–14: *tetB*.

**Fig 3:**
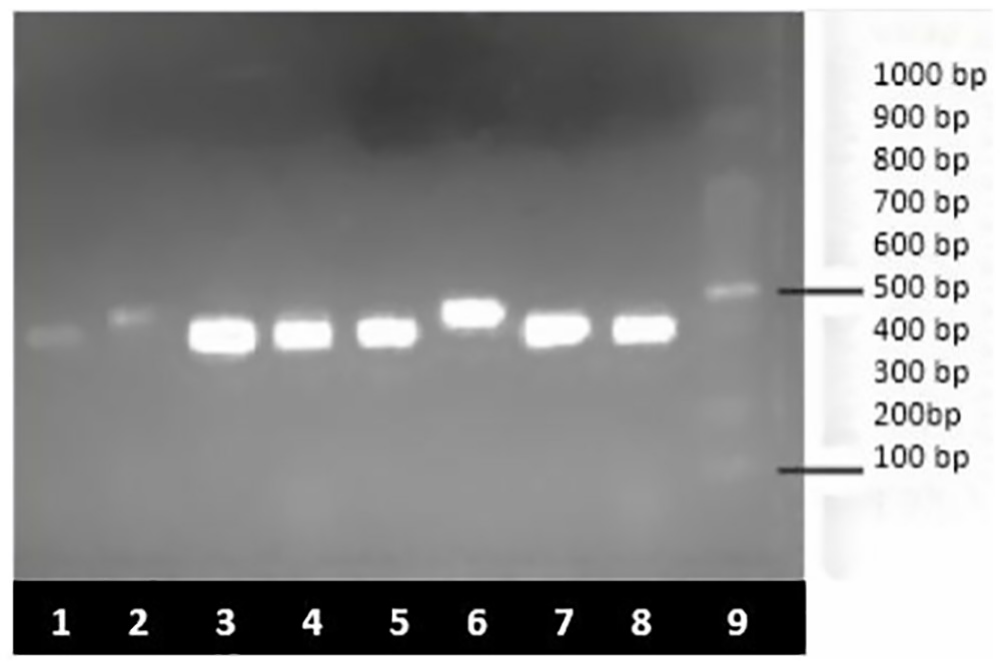
PCR for single nucleotide polymorphism (SNP) of fluoroquinolones (*gyrA* and *parC*) on 1.5% agarose gel. Lane: 1, 3–5, 7–8; *parC* (395 bp), lane 2 and 6: *gyrA* (447 bp) and lane 9: molecular weight marker (100 bp).

**Fig 4:**
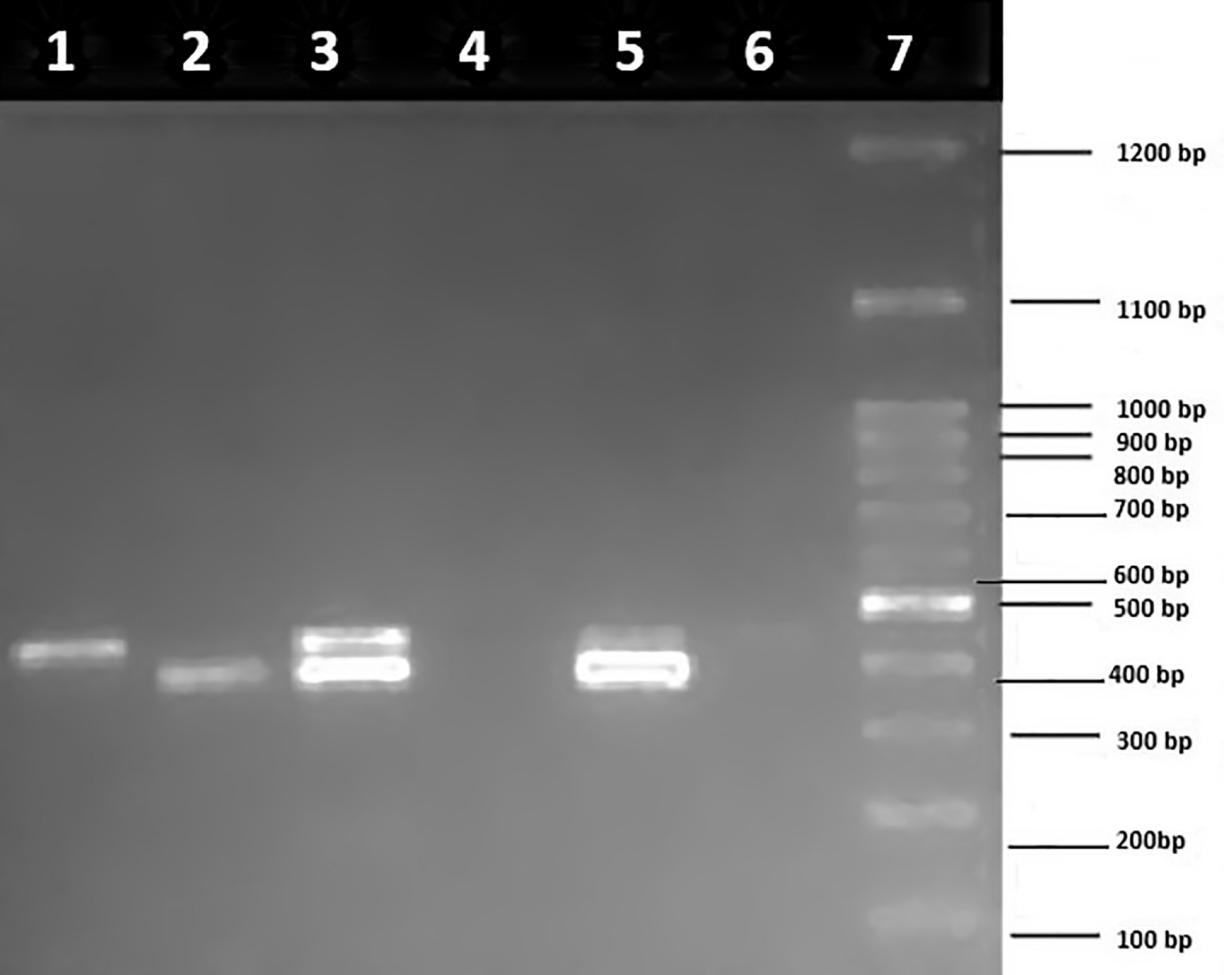
Multiplex PCR for single nucleotide polymorphism (SNP) of fluoroquinolones (*gyrA* and *parC*) on 1.5% agarose gels. Lane 1: *gyrA* (447 bp), lane 2: *parC* (395 bp), lane 3: *gyrA + parC*, lane 4: negative control, lane 5: *gyrA + parC*, lane 6: negative control, and lane 7: molecular weight marker (100 bp).

Owing to the scarcity of detailed studies on the prevalence of antimicrobial resistance patterns in paediatric age group in India, screening of diarrheagenic *E*. *coli* for the presence of virulence genes and drug resistance genes was performed in our previous study [48]. Multiplex PCR for antibiotic resistance genes showed presence of *tetA*, *sul1* and *AacC1* as shown in Fig 1, and *tetB and tetC as shown in* Fig 2. PCR for SNP targeting QRDR of fluoroquinolones (*gyrA* and *parC*) was also performed (Figs 3 and 4).

### Detection of genes conferring resistance to β-lactamases, tetracycline, gentamicin, and sulphonamide

The distribution of various antibiotic resistance genes in three subject groups analyzed by multiplex PCR is shown in Table 2. High frequency of resistance was detected against ampicillin, sulfonamides and tetracycline. Briefly, Significant antibiotic resistance gene frequency was detected in case of *CMY* (15%), *aacC1* (19.16%) and *gyrA + parC* (2.5%). Sul1 gene was found prevalent 57 (47.5%) followed by *TEM*, *SHV*, *tetA and aacC1* (Table 2). Tetracycline resistance gene *tetA* and class 1 integrons usually share the same conjugative plasmid [62]. The prevalence of *sul1* gene in our integron-positive healthy isolates was 47.5% higher than earlier published report [63] suggesting that commensal strains could also harbor these resistance determinants. A significant association between resistance to aminoglycosides tested (gentamicin) and the presence of integron indicate the presence of aminoglycoside resistance genes within integron structures, including *aadA* and *aacA1* [64].

**Table 2:** Distribution of various antibiotic resistance genes in three groups.

Genes	Group-1 n (%)	Group-2 n (%)	Group-3 n (%)	Total n (%)	*p*-value
*TEM*	19(47.5)	14(35)	16(40)	49 (40.83)	0.519
*SHV*	14(35)	14(35)	11(27.5)	39 (32.5)	0.710
*CTX-M*	7(17.5)	8(20)	8(20)	23(19.16)	0.947
*OXA*	9 (22.5)	7 (17.5)	7 (17.5)	23 (19.16)	0.806
*NDM-1*	11 (27.5)	5 (12.5)	6 (15)	22(18.33)	0.178
*IMP*	12 (32.5)	5 (12.5)	6 (15)	23(19.16)	0.098
*VIM*	8 (20)	7 (17.5)	7 (17.5)	22(18.33)	0.945
*ACT*	6 (15)	8 (20)	6 (15)	20 (16.66)	0.786
*DHA*	4 (10)	2 (5)	3 (7.5)	9 (7.5)	0.697
*CMY*	11 (27.5)	4 (10)	3 (7.5)	18 (15)	0.024*
*sul1*	22 (55)	16 (40)	19 (47.5)	57 (47.5)	0.405
*tetA*	13 (32.5)	6 (15)	5 (12.5)	24 (20)	0.051
*aacC1*	12 (30)	9 (22.5)	2 (5)	23 (19.16)	0.014*
*tetB*	5 (12.5)	4 (10)	3 (7.5)	12 (10)	0.757
*tetC*	6 (20)	5 (12.5)	1 (2.5)	12 (10)	0.143
*gyrA*	6 (15)	4 (10)	4 (10)	14 (11.66)	0.723
*parC*	2 (5)	1 (2.5)	0	3 (2.5)	0.358
*gyrA + parC*	3 (7.5)	0	0	3 (2.5)	0.046*

*significant *p*-value. Gene frequencies are present as absolute numbers with percentage in parentheses

SNPs in *gyrA* (A660-T660) and *parC* (C330-T330) were detected in 11.66% and 2.5% isolates, respectively. Among all Nalidixic acid and Ciprofloxacin resistant isolates, 29.78% isolates showed point mutation for *gyrA* gene, while 27.27% isolates showed point mutation for *parC* and 5.17% isolates showed mutation for both *gyrA* and *parC*. However, no mutation was detected in QRDR of *gyrA* and *parC* in 28/48 (58.33%) and 5/11 (45.45%) isolates, respectively; although these isolates were found resistant against Nalidixic acid and Ciprofloxacin, phenotypically {S1 and S2 Tables}.

A multiple logistic regression model was prepared to detect certain independent predictors of antibiotic resistance in three groups (Table 3). It was observed that all independent predictors of antibiotic-resistant genes except *sul1* showed strong association with development of antibiotic resistance (*p*-value < 0.05). Coefficient of adjusted odds ratio was 0.819 times higher in *sul1* as compared to other genes for which it was in the range of 0.004–0.477.

**Table 3:** Multiple logistic regression models exploring certain independent predictors of antibiotic resistance.

Predictors of antibiotic resistance genes	n = 120	p-value	Adjusted Odds ratio	95% CI (Lower)	95% CI (Upper)
**Sul1**					
Present	57	0.438	0.819	0.492	1.362
Absent	63		1		
**TetA**					
Present	24	0.000*	0.063	0.033	0.118
Absent	96		1		
**TetB**					
Present	12	0.000*	0.60	0.24	1.48
Absent	108		1		
**TetC**					
Present	12	0.000*	0.012	0.005	0.028
Absent	108		1		
**aaCa**					
Present	23	0.000*	0.057	0.029	0.107
Absent	97		1		
**TEM**					
Present	49	0.004*	0.477	0.284	0.798
Absent	71		1		
**SHV**					
Present	39	0.000*	0.227	0.131	0.389
Absent	81		1		
**CTX**					
Present	23	0.000*	0.057	0.029	0.107
Absent	97		1		
**OXA**					
Present	23	0.000*	0.057	0.029	0.107
Absent	97		1		
**NDM-1**					
Present	22	0.000*	0.031	0.015	0.063
Absent	98		1		
**IMP**					
Present	23	0.000*	0.031	0.015	0.063
Absent	97		1		
**VIM**					
Present	22	0.000*	0.027	0.013	0.056
Absent	98		1		
**ACT**					
Present	20	0.000*	0.021	0.009	0.044
Absent	100		1		
**CMY**					
Present	18	0.000*	0.021	0.009	0.044
Absent	102		1		
**DHA**					
Present	9	0.000*	0.004	0.001	0.011
Absent	111		1		

*statistically significant

Note: The y variable is antibiotic resistance whereas the variables mentioned as predictors are the independent (x) variables in the multiple logistic regression models.

### 16SrRNA sequencing

**Fig 5:**
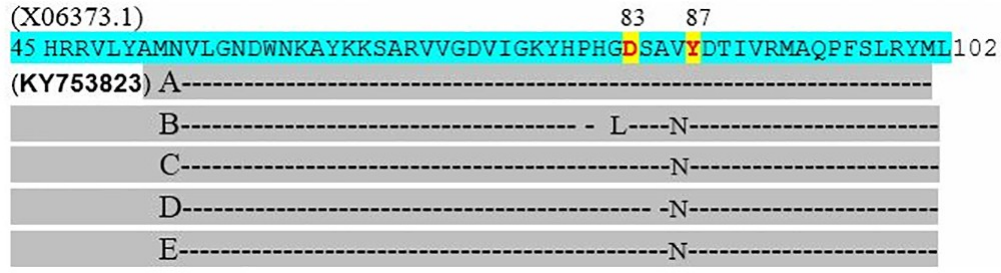
Sequence alignment of the *gyrA* (DNA gyrase) gene sequence (5) that confers resistance to quinolones (Nalidixic acid). Quinolone resistance determining regions (QRDR) were amplified by PCR and sequenced using the primers [56, 57]. The substitution was seen at position 83 (confers high-level resistance) and 87 (confers low-level resistance) as described by [55, 63]. This amino substitution does not alter the stereochemical structure greatly and is therefore unlikely to confer resistance to quinolones on its own. The *gyrA* reference sequence (X06373.1) was obtained from the NCBI database and accession number KY753823 was generated from the study.

**Fig 6:**
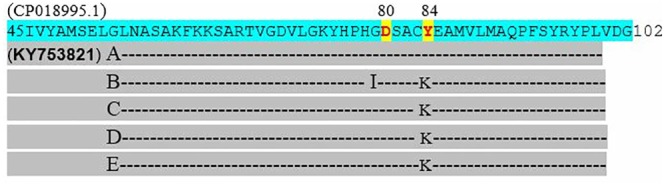
Sequence alignment of the *parC* (DNA topoisomerase) gene sequence (5) that confers resistance to quinolones (Ciprofloxacin). Quinolone resistance determining regions (QRDR) were amplified by PCR and sequenced using the primers [55, 56]. The substitution was seen at position 80 (responsible for quinolone resistance) and 84 (increase affinity to ciprofloxacin by producing positively charged amino acid) as described by [55, 63]. This amino substitution does not alter the stereochemical structure greatly and is therefore unlikely to confer resistance to quinolones on its own. The *parC* reference sequence (CP018995.1) was obtained from the NCBI database and accession number **KY753821** was generated from the study.

Few positive isolates were sequenced and submitted to NCBI (accession numbers: *gyrA* KY753823 and *parC* KY753821). Alignment of the *gyrA* (DNA *GyrA*se) gene sequence conferring resistance to Nalidixic acid and alignment of the *parC* (DNA topoisomerase) gene sequence conferring resistance to Ciprofloxacin is shown in Figs 5 and 6 [55–57,65,66]. The 16SrRNA was amplified using universal primers and the nucleotide sequences of the 16SrRNA of all the isolates were submitted to NCBI and following accession numbers were obtained: KY775448, KY775449, KY786039, KY786040, KY786041, KY786042, KY786043, KY786044, KY786045, KY786046, and KY786047.

### Phylogenetic analysis

**Fig 7:**
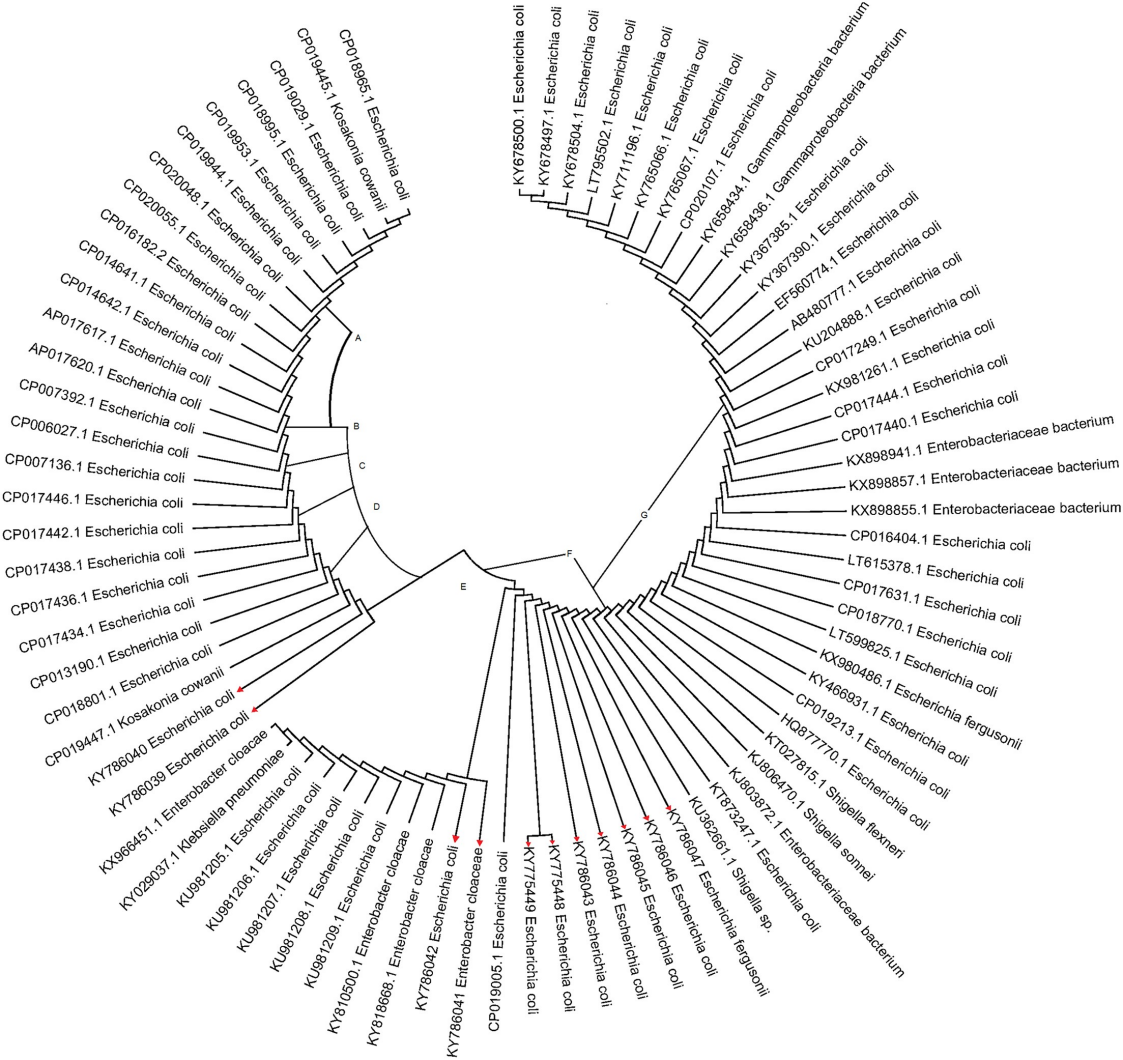
Phylogenetic analysis based on the sequences of 16SrRNA gene sequence of 11 diarrheagenic *E*. *coli* (DEC) isolates and 70 sequences retrieved from NCBI. The accession number of each strain is mentioned in the tree. Eleven isolates identified by this study are highlighted in red.

Eleven isolates from the current study were used to construct a phylogenetic tree along with other sequences from database for 16SrRNA as described in detail previously by Shashi and Kumar [67] (Fig 7). Significant diversity was evident among these isolates. The phylogenetic tree showed that all the isolates could be grouped into seven phylogroups on the basis of approximately 98% similarity among them (Fig 7). Briefly, the prevalence of phylogenetic group B2 was 36.66%, followed by groups B1, A, F, D, E and C in our study (Table 4). Similar pattern was observed in the healthy isolates with the prevalence of group B2, followed by B1, F and A. All the isolates were assigned a phylogroup except 11 isolates which remained unclassified. None of the isolates recovered from diarrheagenic cases or healthy controls belonged to phylogroup Clade I.

**Table 4:** Distribution of various phylogenetic groups in three study populations.

Phylogroup	Group-1 n (%)	Group-2 n (%)	Group-3 n (%)	Total n (%)	*p*-value
*A*	7 (17.5)	5 (12.5)	7 (17.5)	19 (15.83)	0.778
*B1*	9 (22.5)	6 (15)	11 (27.5)	26 (21.66)	0.393
*B2*	17 (4/2.5)	15 (37.5)	12 (30)	44 (36.66)	0.505
*C*	1 (2.5)	1 (2.5)	1 (2.5)	3 (2.5)	1.000
*D*	3 (7.5)	1 (2.5)	2 (5)	6 (5)	0.590
*E*	1 (2.5)	1 (2.5)	2 (5)	4 (3.33)	0.772
*F*	3 (7.5)	3 (7.5)	2 (5)	8 (6.66)	0.874
*Clade 1*	0	0	0	0	N.A
*Unclassified*	4 (10)	4 (10)	3 (7.5)	11 (9.16)	0.904

Gene frequencies are present as absolute numbers with percentage in parentheses

## Discussion

Antibiotic resistance in bacteria is not only a serious global health problem worldwide but also it renders most of the antibiotics ineffective. The threat is compounded with the continuous spread of drug resistance and enhanced survival potential of such bacterial strains [68, 69]. Since, changes at the gene level like compensatory or suppressor mutations may favor the survival of resistant microbe, the selection of the most virulent and resistant pathogens depends upon the antimicrobial selective pressure [69, 70]. We attempted to analyze the acquisition of point mutations in *gyrA* and *parC* genes in QRDR; tetracycline, sulphonamide and gentamicin resistance genes in isolates of *E*. *coli*. Tetracycline resistance gene *tetA* and class 1 integrons are normally present on the same conjugative plasmid [63], but the acquisition of gentamicin resistance genes is unclear. The prevalence of *sul1* gene in isolates recovered from healthy subjects samples was 47.5% higher when compared with an early report, suggesting that the commensal strains may also transfer these resistance determinants to neighboring susceptible commensals [64].

Enzymes DNA *gyrase*, encoded by *gyrA* and gyrB, and DNA topoisomerase, encoded by *parC* and *parE*, are important for bacterial replication and thus primary targets of quinolones. Mutation in the *parC* gene confers resistance to the secondary class of fluoroquinolones. We observed less prevalence of *parC* mutation in fluoroquinolones/ciprofloxacin resistant isolates in our population, in contrast to earlier reports [42, 71–74].

An SNP prevalence of 11.66% and 2.5% isolates was detected in *gyrA* and *parC* gene respectively, corroborating the findings of previous studies [33, 42, 70, 75–77]. Interestingly, most of the ESBL-producing isolates in our study were resistant to ciprofloxacin having mutations in *gyrA*/*parC* genes demonstrating underlying causes of fluoroquinolone resistance [78–80].

It is well-known that *E*. *coli* is no longer restricted to the hospital environment [81]. The β-lactamase genes harboring MDR strains are found in healthy children raising an underlying threat of widespread circulation of resistant strains in the community [82, 83]. The genes located on transmissible plasmid along with other antibiotic resistance genes enables an easy dissemination in the environment and amongst hospitalized patients [84]. The *VIM* encoding integron structure acquired during the hospital stay may also colonize in patients and retain as reservoirs [85]. The emergence of the *CMY* gene has also been reported in *E*. *coli* along with other diverse genera of the *Enterobacteriaceae* [86]. Several other factors like overcrowding, availability of antibiotics, low level of hygiene and weak hospital antibiotic policies are also responsible for their extensive clonal dissemination [87].

All antibiotic resistant genes, except sulphonamide, appear as important predictors of drug resistance in paediatric population. Low frequency of occurrence of genes of tetracycline, aminoglycosides and other β-lactamases genes perform well as indicators of emerging resistance in children, unlike the sulphonamide resistance which was uniform in all the study groups. Majority of the ESBLs found in *E*. *coli* are derivatives of *TEM* or *SHV* enzymes while *CTX-M* and *OXA*-type beta-lactamase occur less frequently [88, 89].

The ABLs (AmpC β-lactamases) is one of the prevalent mechanisms of β-lactam resistance after ESBLs in *E*. *coli* and emerged as an important health problem in the recent years [90, 91]. There are various factors which are associated with development of quinolone resistance, including chromosomal mutations, acquisition of plasmid-mediated genes and decreased uptake of the antimicrobials [92]. We observed occurrence of multiple transferrable resistance genes in *E coli*. This bacteria being an essential gut microflora, may facilitate the promulgation of resistance determinants to other microbiome and its prolonged survival helps create a huge reservoir of drug resistant microbes [93].

The *E*. *coli* phylogroups have different ecological niches, biological characteristics and ability to cause disease. Early reports suggested a link between phylogeny and virulence determinants [94], that are often carried by strains of phylogenetic groups B2 and D [95, 96]. Due to the small number of subjects, the phylogenetic analysis of the isolates did not show any significant difference between the phylogroups. Variations in environmental conditions and host genetic factors may be the responsible for the contrasting findings from other reports [97, 98]. Our results showed the preponderance of phylogenetic group B2 (36.66%) similar to previous reports [97, 98]. Groups B1, A, F, D, E and C were found to have 21.66%, 15.83%, 5.83%, 5%, 3.33% and 2.5% isolates, respectively. We also found commensal phylogroups A and B1 in agreement with previous studies showing that diarrheagenic *E*. *coli* isolates are included in phylogroups A, B1, and D [99, 100]. A total of 11 isolates (9.16%) remained unidentified as they were negative for all the genes by quadruplex PCR. The ecological distribution of phylogenetic groups of human *E*. *coli* isolates are thus variable and dynamic, influenced by factors such as host genetic makeup, dietary conditions, use of medications, and geographical circumstances often useful in describing the profile of the particular community [82, 83].

We could also reveal 5% isolates associated with phylogroup D that is linked with the spread of AmpC- mediated antibiotic resistance (especially *CMY*-2 type) [101–104]. Further, this group is also involved in the spread of *CTX-M* genes [105–107]. The existence of more than 40% of our isolates under phylogroups B2 and D is worth noting as these are associated with ESBLs and AmpCs expressing *E*. *coli* strains linked with higher virulence characteristics as described in early studies on phylogroups A and B1 [103, 108, 109]. Although, little is known about the association of MBL resistance with phylogroups in *E*. *coli*, phylogroups B1 and D are thought to be associated with *NDM*-1 type [110–114]. The study highlights that children harbor pathogenic as well as commensal strains of *E*. *coli* in alarming abundance and their co-existence in similar niches enable them to maintain a continuous circulation of gene transfer. This observation draws attention to an urgent need for preventing future catastrophe. Bacterial populations in the human gut are complex and share a similar ecology, giving them abundant opportunity for the transfer of genetic material [115].

The current scientific advances have created a wide area of interest amongst scientists to understand the spread of antibiotic resistance genes, and the field of metagenomics have enabled them to create a database of gut commensal resistome from healthy individuals from different countries. Documented evidences state that countries with relatively reserved policies of antibiotic use in humans and animals (like Denmark) have observed lower levels of antibiotic resistance genes in human gut microbiota than in people from countries where antibiotic use is considerably higher (like Spain and China) [116]. Therefore, it is high time to raise awareness amongst health care providers and develop country wise national policies for rationale use of antibiotics in humans especially amongst the vulnerable pediatric population to combat the menace of drug resistance [116, 117].

### Conclusion

The spread of antimicrobial resistance has emerged as an important public health problem especially in resource limited countries where lack of strict adherence to antibiotic policy has created a challenge for the clinicians to treat serious infections essentially in prolonged hospitalized patients. Our phylogenetic analysis identified 40% of the isolates grouped as B2 and D which mostly harbor ESBL and ABL expressing *E*. *coli* strains. Mankind has partly been responsible for creating such an environment for the microbial world to develop armamentarium for challenging the antimicrobial agents. Gut flora is the first line of defense, and harboring drug-resistant pathogens will be detrimental not only to that individual but will be a threat to the community. Antibiotic resistance has extended from hospital to community settings as well, suggesting that healthy children may also contribute to the development of MDR in *E*. *coli*. Our observation in pediatric population is a grim reality to the development, dissemination and carriage of antibiotic resistant bugs not only in the gut of diarrhoeal children but also in healthy children of our community. Active AMR surveillance and stewardship programs needs to be implemented in all hospitals to minimize further danger.

## Supporting information

S1 Fig Trends in antibiotic consumption in India from the year 2000 to 2015. The data used to create this figure can be accessed at the Center for Disease Dynamics, Economics & Policy (CDDEP) Resistance Map website at http://resistancemap.cddep.org/resmap/c/in/India.
(PDF)

S1 Table Details of isolates in the three groups. (PDF)

S2 Table Frequency of resistance to antimicrobial agents of *E*. *coli* isolates from the three study groups. (PDF)
