# Supplementary material for: Changing paradigm of antibiotic resistance amongst Escherichia coli isolates in Indian pediatric population
Source: PLoS One. 2019 Apr 17;14(4):e0213850. doi: 10.1371/journal.pone.0213850 (PMC6469777; doi:10.1371/journal.pone.0213850)
Supplement: S1 Fig — The data used to create this figure can be accessed at the Center for Disease Dynamics, Economics & Policy (CDDEP) Resistance Map website at http://resistancemap.cddep.org/resmap/c/in/India. (PDF) [file pone.0213850.s001.pdf]

S1 Fig: Trends in antibiotic consumption in India from the year 2000 to 2015. The data used to create this figure can be accessed at the Center for Disease Dynamics, Economics & Policy (CDDEP) Resistance Map website at http://resistancemap.cddep.org/resmap/c/in/India.

(A)

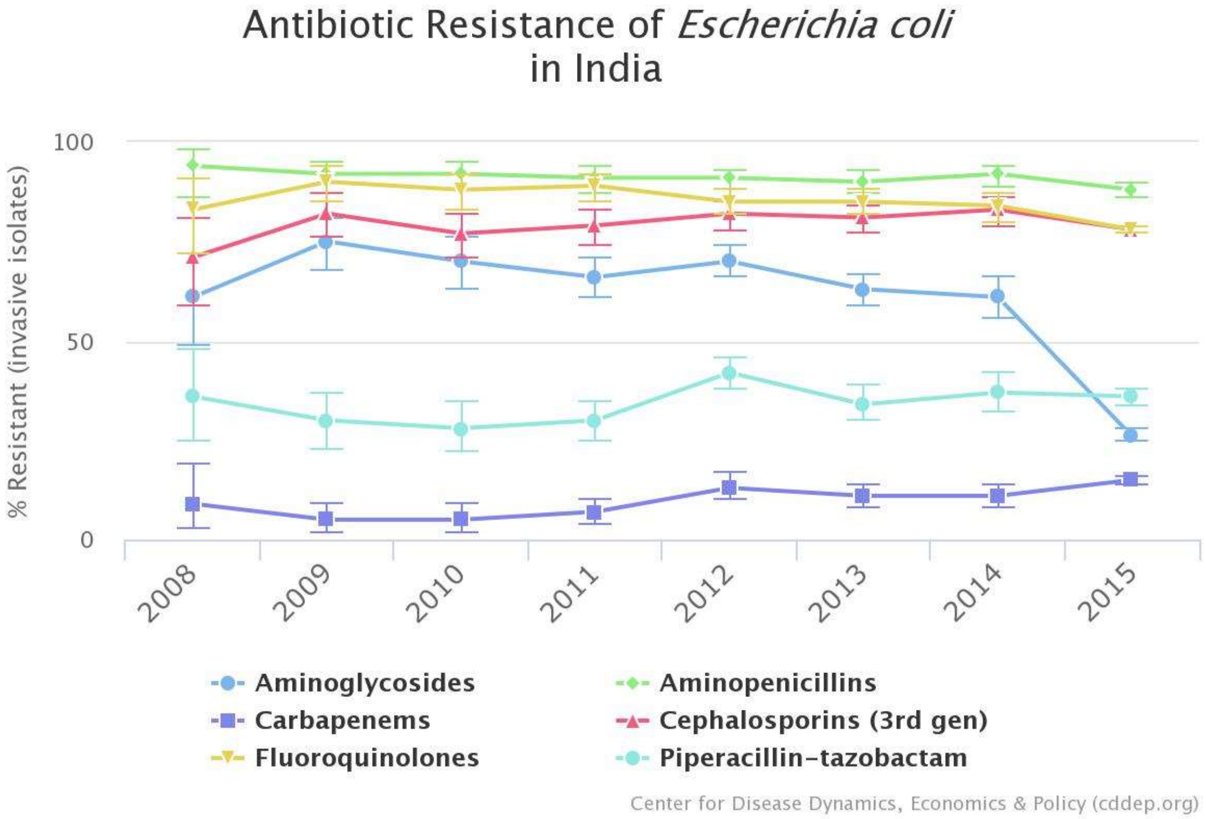

(B)

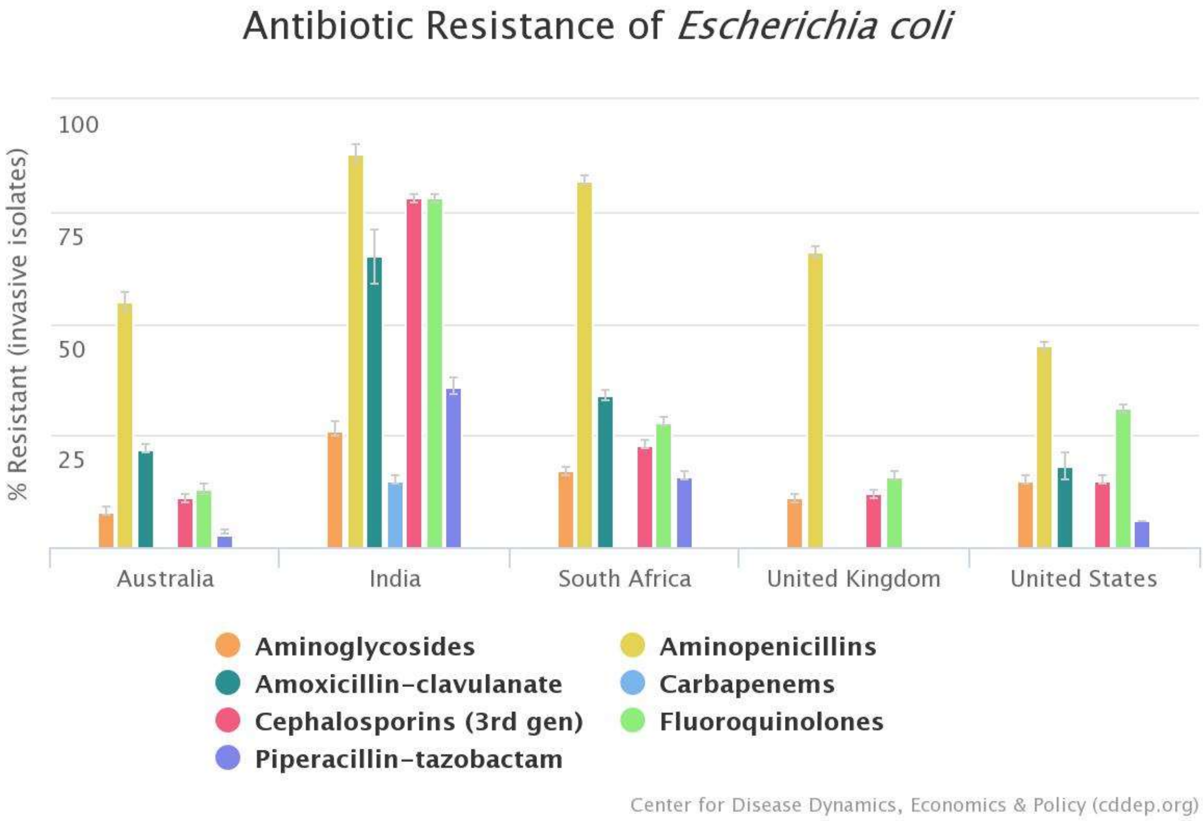
Antibiotic Resistance of *Escherichia coli*
100
75
50
25
% Resistant (invasive isolates)
Australia
India
South Africa
United Kingdom
United States
Aminoglycosides
Aminopenicillins
Amoxicillin-clavulanate
Carbapenems
Cephalosporins (3rd gen)
Fluoroquinolones
Piperacillin-tazobactam
Center for Disease Dynamics, Economics & Policy (cddep.org)

(C)

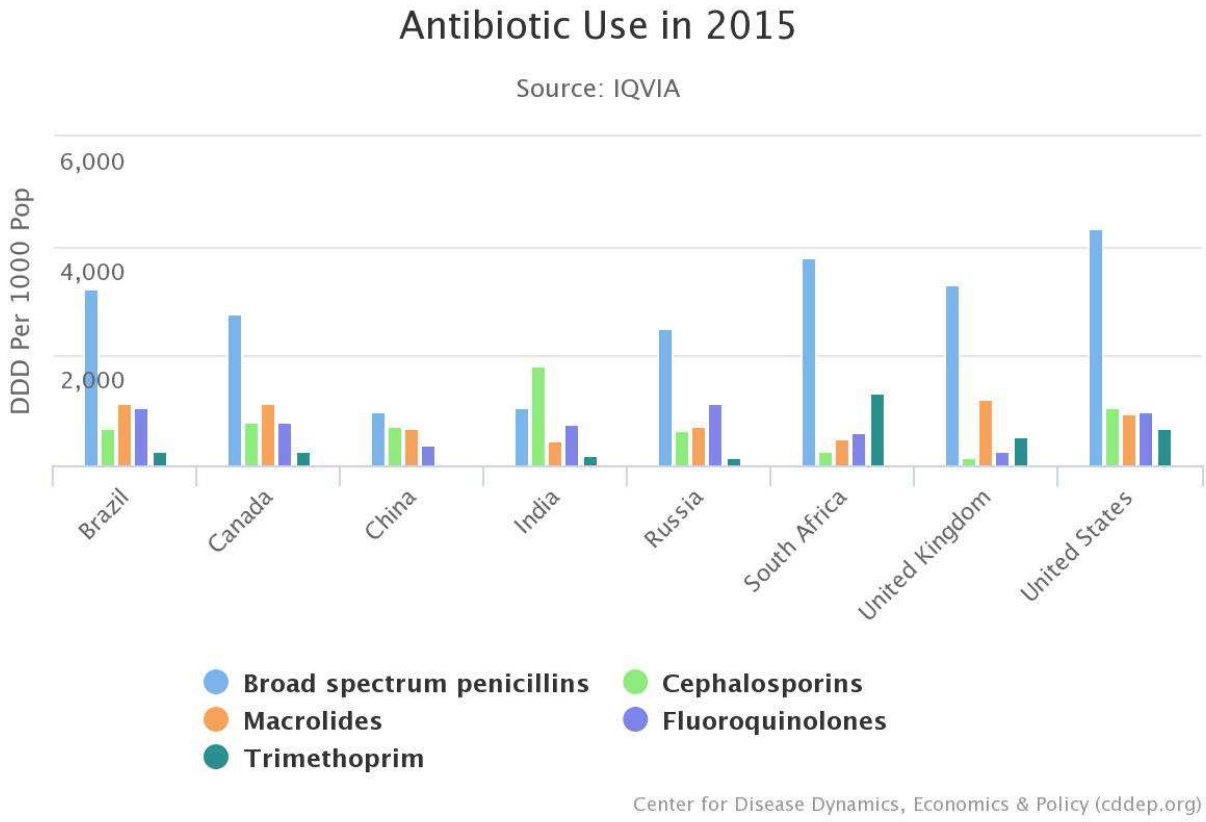

Antibiotic Use in 2015
Source: IQVIA
DDD Per 1000 Pop
6,000
4,000
2,000
Brazil
Canada
China
India
Russia
South Africa
United Kingdom
United States
Broad spectrum penicillins
Cephalosporins
Macrolides
Fluoroquinolones
Trimethoprim
Center for Disease Dynamics, Economics & Policy (cddep.org)

(D)

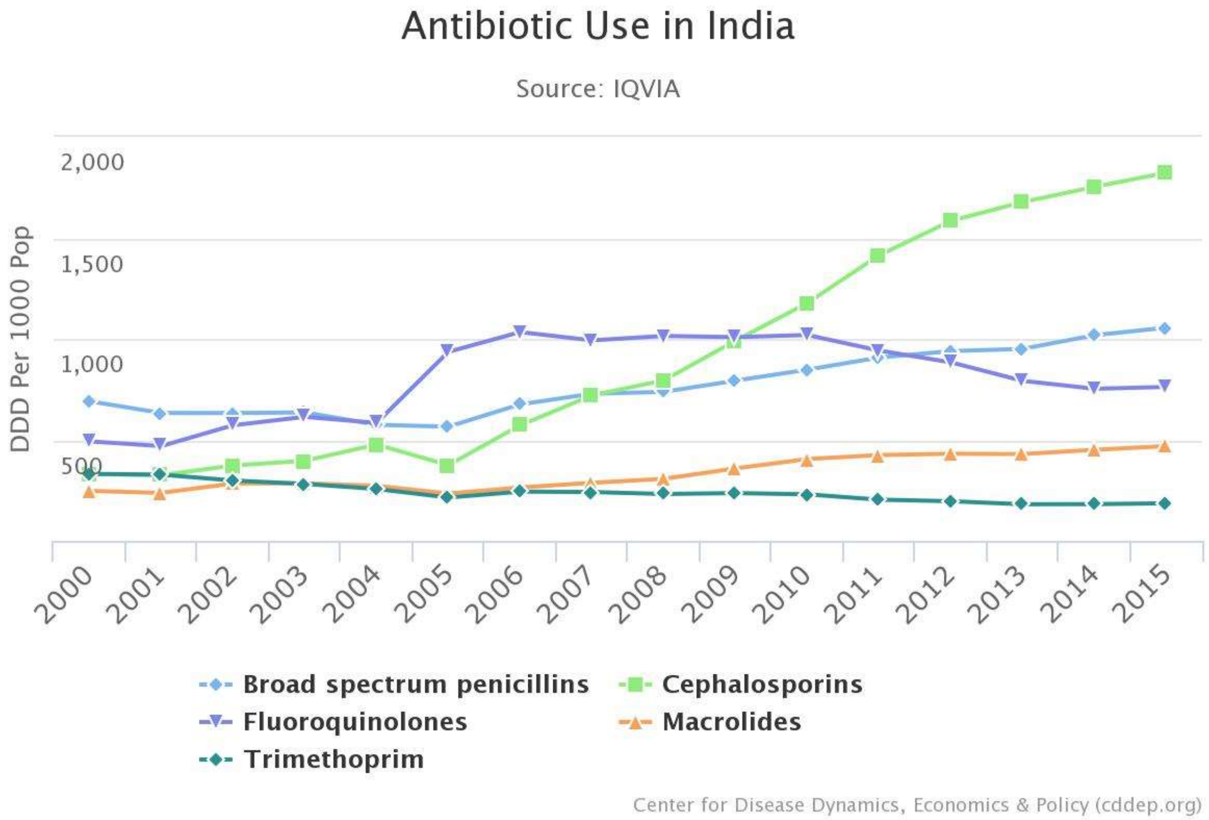

Antibiotic Use in India
Source: IQVIA
DDD Per 1000 Pop
2,000
1,500
1,000
500
2000
2001
2002
2003
2004
2005
2006
2007
2008
2009
2010
2011
2012
2013
2014
2015
Broad spectrum penicillins
Cephalosporins
Fluoroquinolones
Macrolides
Trimethoprim
Center for Disease Dynamics, Economics & Policy (cddep.org)
