# Supplementary material for: Changing paradigm of antibiotic resistance amongst Escherichia coli isolates in Indian pediatric population
Source: PLoS One. 2019 Apr 17;14(4):e0213850. doi: 10.1371/journal.pone.0213850 (PMC6469777; doi:10.1371/journal.pone.0213850)
Supplement: S1 Table — (PDF) [file pone.0213850.s002.pdf]

**S1 Table:** Details of isolates in the three groups.

| S.No. | Age | Sex | Beta-lactamase phenotype | Beta-lactamase genes detected | Resistance phenotype to antibiotics | Virulence genes detected | Phylogroups detected | Antibiotic resistance genes detected | SNP mutation gyrA/parC | Position(s) of amino acid change(s) in: GyrA ParC |
|---|---|---|---|---|---|---|---|---|---|---|
| 1O | 3-5 years | female | ESBL | Tem, shv | NF-CTX-NA-CIP-AMK | Bfp, elt, est, eae, eaf, eagg | B1,B2 | sul1 | gyrA + parC | 83 80 |
| 2O | 0-1 years | female | ESBL | Act, dha, tem, | CTX | Bfp, stx, hyla | A | tetA, tetC | None | |
| 3O | 0-1 years | Male | ESBL | Tem | CTX | Bfp, est, eaf, eagg | NONE | tetA, tetB | None | |
| 4O | 1- 3 years | female | ESBL | Shv, ctx-m | CTX-NA-TZP | Bfp, est, eaf, eagg | F | sul1 | GyrA | 83+87 |
| 5O | 3-5 years | Female | ESBL | Act, cmy, tem, oxa | IPM-MEM-AZT-GEN-AMP-AMK | Bfp, eaf, eagg | B2 | AacC1 | None | |
| 6O | 0-1 years | Male | ESBL | Ctx | NF-CAZ-NA | Bfp, est, eae, eaf, eagg | B1, E | sul1 | None | |
| 7O | 1- 3 years | Female | AMPC, AmpC | Vim, act | NF-CTX-NA-GEN-CIP | Bfp, elt, est, eagg | A | sul1, AacC1 | gyrA + parC | 83+87, 80 |
| 8O | 0-1 years | Male | ESBL | Tem | CTX-CIP-PB-TZP | Stx | B2 | sul1 | ParC | 80 |
| 9O | 1- 3 years | Male | ESBL | Shv, oxa | CTX-CAZ-GEN-NA-AMK | Bfp, east | B2 | sul1, AacC1, | None | |
| 10O | 1- 3 years | Male | NONE | Shv | GEN-TZP | Bfp, est, eae, eaf, eagg | D | AacC1 | None | |
| 11O | 0-1 years | Male | ESBL | Oxa | AZT | Bfp, est, eae, eagg, east | B1, B2 | None | None | |
| 12O | 1- 3 years | Female | NONE | None | CTX-IPM-NA-AMK | Bfp | B2 | sul1 | GyrA | 83 |
| 13O | 0-1 years | Male | AmpC | Cmy | CTX-CAZ-GEN-NA-AMK | East | A | sul1, AacC1 | None | |
| 14O | 0-1 years | Male | NONE | None | CTX-IPM-MEM-CAZ-TZP | Bfp, est, eaf, eagg, east, stx, hyla | B2 | tetA, tetC | None | |
| 15O | 0-1 years | Male | ESBL | Cmy, tem, shv, ctx | NF-NA-TZP | Bfp, est | B1 | sul1 | GyrA | 83 |
| 16O | 3-5 years | Male | ESBL | Tem, shv | CTX-NA-GEN-CIP-TZP | Est, eagg | A | tetA, sul1, AacC1, tetC | gyrA + parC | 83 80+84 |
| 17O | 3-5 years | Male | AmpC | Act,cmy | CTX-CIP-AMK | Elt, eae | B1, B2, C | sul1 | None | |
| 18O | 0-1 years | Female | NONE | None | CIP | Elt, eaf, eagg | NONE | tetA,sul1, tetB | None | |
| 19O | 0-1 years | Male | ESBL | Dha, cmy, tem, oxa | AZT | Bfp, est, eagg | NONE | tetA, tetB | None | |
| 20O | 1- 3 years | Female | ESBL | Tem | GEN-NA | None | B1, F | sul1, AacC1 | None | |
| 21O | 1- 3 years | Female | NONE | Tem, shv | NF-CTX-NA-AMP-TZP | East, stx, hyla | B2 | sul1 | None | |
| 22O | 0-1 years | Male | ESBL | Tem, ctx | CTX-CAZ-AMK | Bfp, est, eae, eaf, eagg | A | TetA | None | |
| 23O | 3-5 years | Male | ESBL | Ctx | CTX | Bfp, est, eagg | B1 | tetA, tetC | None | |
| 24O | 1- 3 years | Female | ESBL | Act, tem, shv, oxa | NF-CTX-IPM | Elt, eagg | A, B1 | sul1 | None | |
| 25O | 1- 3 years | Female | ESBL,AMPC | Vim, tem, shv | CTX-AMC-GEN-AMK-TZP | Elt, est, eae, eagg, east | B2 | tetA, AacC1, tetC | None | |
| 26O | 0-1 years | Male | AmpC | Act, dha, shv | CTX | Bfp, elt, eaf, eagg | NONE | None | None | |
| 27O | 1- 3 years | Male | AMPC | Imp, vim | NF-CTX-CAZ-AZT-NA | Bfp, est | A, B1, B2 | sul1 | GyrA | 83+87 |
| 28O | 1- 3 years | Male | NONE | Shv | CIP-AMK | Bfp, est,eagg | B1, B2, E | tetA, tetB | ParC | 80+84 |
| 29O | 0-1 years | Female | ESBL | Tem | CTX-GEN | Eaf, eagg | NONE | AacC1 | None | |
| 30O | 0-1 years | Female | ESBL | Tem, shv, ctx | GEN | Bfp, est, eagg, east | B2 | tetA, AacC1 | None | |
| 31O | 1- 3 years | Female | ESBL | Tem, ctx | NA-TZP | Elt, eagg | B2 | sul1 | None | |
| 32O | 0-1 years | Male | ESBL | None | NF-CTX-GEN | Bfp, est, eagg,stx, hyla | B2 | sul1, AacC1 | None | |
| 33O | 0-1 years | Male | AMPC, AmpC | Imp, dha, cmy, tem | CTX-IPM-CAZ | Eagg | A | None | None | |
| 34O | 0-1 years | Male | ESBL | Ndm, vim | CTX-NA | Elt, eaf | B1, B2 | sul1 | None | |
| 35O | 0-1 years | Male | ESBL | None | CTX | Bfp, est | B2 | tetA, tetB | None | |

| | | | | | | | | | | |
|---|---|---|---|---|---|---|---|---|---|---|
| 36O | 1- 3 years | Male | AmpC | Act | GEN-TZP | Est, east | A, B2 | AacC1 | None | |
| 37O | 1- 3 years | Female | ESBL | None | NF-NA | Bfp, est, eae, eaf, eagg | B2 | sul1 | None | |
| 38O | 1- 3 years | Male | ESBL | Tem, shv | CTX-CIP | Bfp, est, eae, eaf, eagg | B1, F | tetA, sul1, tetC | None | |
| 39O | 0-1 years | Female | ESBL | None | CTX-CAZ-NA-GEN | Bfp, elt, east | B2 | sul1, AacC1 | GyrA | 83 |
| 40O | 0-1 years | Male | ESBL | Tem, oxa | CTX-AZT-NA-GEN-CIP | Bfp, elt, est, east | B2 | sul1, AacC1 | GyrA | 83 |
| 1I | 1- 3 years | Male | ESBL | Act, cmy | CTX-AZT-NA-TZP | Eagg, east | NONE | tetA, sul1, tetB | None | |
| 2I | 0-1 years | Male | ESBL | Tem | CTX-AMK | Bfp, elt, est, eaf, eagg, east | NONE | None | None | |
| 3I | 0-1 years | Male | ESBL | Imp, vim, tem, shv | CIP | Elt, est, eagg, east | B1 | sul1 | ParC | 80 |
| 4I | 1- 3 years | Female | NONE | None | GEN | Elt, est, eaf, eagg, east | A | AacC1 | None | |
| 5I | 0-1 years | Female | NONE | Shv | NF-CTX | Eaf, eagg | D | sul1 | None | |
| 6I | 0-1 years | Male | ESBL | Tem, ctx | CTX-GEN-AMK-TZP | None | NONE | AacC1 | None | |
| 7I | 0-1 years | Male | ESBL,AMPC | Vim, shv, oxa | CTX-IPM-NA-AMK-CCA | Bfp, est, eae, eaf, eagg, east | A, B2 | tetA, sul1, tetC | None | |
| 8I | 3-5 years | Male | ESBL,AMPC | Ndm,vim, shv, oxa | CTX-GEN | Eaf, east | B1, B2, F | AacC1 | None | |
| 9I | 1- 3 years | Male | ESBL | None | CTX-AZT-GEN-TZP | East | NONE | AacC1 | None | |
| 10I | 1- 3 years | Female | ESBL | Tem, ctx | AZT-NA-AMP | Est, eae, eagg, east | NONE | sul1, AacC1 | GyrA | 83 |
| 11I | 0-1 years | Male | AMPC, AmpC | Ndm, imp, act | CTX-AMK | Est, eae, eaf, east | NONE | tetA, tetB | None | |
| 12I | 0-1 years | Female | ESBL,AMPC | Imp, vim, tem | NF-CAZ | Bfp, est, eae, eaf | C | sul1 | None | |
| 13I | 0-1 years | Female | AMPC | Imp, vim | CTX-GEN-CRO | Stx | B2 | AacC1 | None | |
| 14I | 3-5 years | Male | AMPC, AmpC | Act, cmy, shv | NF-CTX-GEN-NA-TZP | Bfp, east, stx | A | sul1, AacC1 | None | |
| 15I | 0-1 years | Male | ESBL,AmpC | Tem, shv | IPM-AMC-AMK-TZP | Est, eae, eaf, east, hyla | B1 | tetA, sul1, AacC1 | None | |
| 16I | 1- 3 years | Male | AMPC | Ndm, vim | CTX-IPM-MEM-CAZ | Bfp | B2 | None | None | |
| 17I | 3-5 years | Female | AMPC | Vim | CTX-AMK | East | NONE | None | None | |
| 18I | 3-5 years | Female | ESBL | Ctx, oxa | CTX-NA-AMK | Bfp, east, hyla | NONE | tetA, sul1, tetC | None | |
| 19I | 0-1 years | Female | ESBL | Tem, shv | CTX-TZP | East | D | None | None | |
| 20I | 0-1 years | Female | ESBL,AMPC | Ndm, tem, shv, ctx | CTX-GEN | Est | A, B2 | AacC1, | None | |
| 21I | 0-1 years | Female | ESBL,AMPC | Ndm, imp, ctx, oxa | CTX-AMP | Eae, eaf, east | B2 | tetA, tetB | None | |
| 22I | 1- 3 years | Female | AMPC | None | CTX-NA-TZP | Bfp, est, eae, eaf | B1 | sul1 | None | |
| 23I | 0-1 years | Male | ESBL | Shv, ctx | NF-CTX-AZT-TZP | Bfp, est, eae | E | sul1 | None | |
| 24I | 0-1 years | Female | NONE | None | CTX-GEN | Est, eae, eaf, eagg | B1 | AacC1 | None | |
| 25I | 3-5 years | Female | ESBL | Act, cmy | CTX | Est, eae, eagg | B2 | tetA, tetC | None | |
| 26I | 0-1 years | Male | NONE | None | NF-CTX-NA-AMK | Bfp, elt, est, east | B1, B2 | sul1 | GyrA | 83+87 |
| 27I | 1- 3 years | Female | NONE | None | NF-CTX-AMP | Bfp, est, eae, eaf, eagg, east | B1 | tetA, sul1 | None | |
| 28I | 0-1 years | Female | ESBL,AMPC | Tem, shv | CTX-AMK-TZP | Est, eae, eaf | A, B2, F | None | None | |
| 29I | 0-1 years | Male | NONE | Act, tem, shv, ctx | CAZ-GEN-NA | Bfp, est, eae, eagg | B2 | sul1, AacC1, | None | |
| 30I | 0-1 years | Male | NONE | None | CTX-NA | Bfp, est, eae, eagg | B1 | tetA, sul1, tetC | None | |
| 31I | 0-1 years | Female | ESBL,AmpC | Tem, shv, ctx | CTX-AMK | Eae, eaf, eagg | B2 | tetA, tetB | None | |
| 32I | 3-5 years | Female | ESBL | Tem, oxa | CTX-GEN-NA | Bfp, elt, eae, eaf, eagg | NONE | sul1, AacC1, | GyrA | 83 |
| 33I | 1- 3 years | Male | ESBL,AMPC | Vim, shv | CTX-AZT-GEN-NA | Bfp, eae, eaf, eagg | B2, F | sul1, AacC1, | GyrA | 83 |

| | | | | | | | | | | |
|---|---|---|---|---|---|---|---|---|---|---|
| 34I | 0-1 years | Male | ESBL | Shv, ctx | CTX-GEN-NA | Eae, eaf | B1 | sul1, AacC1 | None | |
| 35I | 3-5 years | Female | ESBL,AmpC | Dha, tem | CTX-AMK | Elt, eae, eaf, eagg, east | NONE | tetA, tetC | None | |
| 36I | 0-1 years | Female | ESBL | Act | CTX-NA | Bfp, eae, eagg | NONE | sul1 | None | |
| 37I | 3-5 years | Female | ESBL,AmpC | Cmy, ctx, oxa | CTX | Est, eae, eaf, eagg | B2, D | None | None | |
| 38I | 0-1 years | Male | ESBL,AmpC | Dha | CTX-AMC-GEN-NA-AMK | Bfp, elt, eae, eaf | NONE | sul1, AacC1, | None | |
| 39I | 3-5 years | Male | ESBL | Imp, vim, tem, oxa | CTX-GEN-NA | Eae, eaf, eagg | NONE | sul1, AacC1 | None | |
| 40I | 0-1 years | Male | ESBL | Ctx | NF-CAZ-NA-AMK | Bfp, est, eae, eagg | NONE | sul1, dfrB1 | None | |
| 1C | 1- 3 years | Male | NONE | None | NF-GEN-NA | None | A, B2 | sul1, AacC1 | GyrA | 83 |
| 2C | 0-1 years | female | AmpC | Act, shv | NONE | None | NONE | None | None | |
| 3C | 3-5 years | Male | ESBL | Tem, shv | CTX-NA | None | B2 | tetA, sul1, tetB | None | |
| 4C | 3-5 years | Male | ESBL | Tem | NA | None | NONE | sul1, | None | |
| 5C | 3-5 years | Female | ESBL | Shv, ctx, oxa | NA | None | NONE | sul1, | None | |
| 6C | 0-1 years | Male | AMPC,AmpC | Ndm, act, ctx | NA-TZP | None | A | sul1, | None | |
| 7C | 3-5 years | Male | ESBL,AMPC | Imp, vim, act, cmy, tem, shv, oxa | NF-GEN | Eagg | NONE | sul1, AacC1 | None | |
| 8C | 1- 3 years | Male | AMPC | Ndm | NF | Eae | B1, B2 | sul1 | None | |
| 9C | 3-5 years | Male | ESBL | Shv | NF-CIP | Eae | NONE | sul1 | None | |
| 10C | 1- 3 years | Male | NONE | Ctx | NONE | Eae, eagg | A | None | None | |
| 11C | 3-5 years | Male | AmpC | Dha | NA | Bfp, eaf, eagg | NONE | sul 1 | None | |
| 12C | 0-1 years | Male | ESBL | Tem, shv, oxa | CTX-NA-CRO | Stx, hyla | B1, B2 | tetA, sul1 | GyrA | 83+87 |
| 13C | 0-1 years | Male | ESBL | Ctx | CTX-NA | None | NONE | sul1 | None | |
| 14C | 0-1 years | Male | ESBL | Shv, oxa | NONE | None | NONE | None | None | |
| 15C | 1- 3 years | Female | AMPC,AmpC | Vim, cmy, ctx | NONE | Bfp | B2, C | None | None | |
| 16C | 0-1 years | Male | ESBL,AMPC | Ndm, vim, tem, oxa | NA | bfp | NONE | sul1 | None | |
| 17C | 0-1 years | Male | AMPC | Imp | NONE | Eae | B1 | None | None | |
| 18C | 3-5 years | Male | ESBL | Tem | NA | Eae | NONE | None | None | |
| 19C | 1- 3 years | Male | NONE | None | NA | Eaf | E | sul1 | None | |
| 20C | 1- 3 years | Male | AmpC | Act, cmy | NONE | Eagg | NONE | None | None | |
| 21C | 1- 3 years | Female | ESBL | Tem | CTX | East | B2 | tetA, tetB | None | |
| 22C | 0-1 years | Male | ESBL | Shv | NA | Eaf, eagg | NONE | sul1 | GyrA | 83 |
| 23C | 1- 3 years | Male | ESBL | Act, dha, tem, shv | NF | Bfp, east | NONE | sul1 | None | |
| 24C | 3-5 years | Male | AMPC,AmpC | Ndm, vim, act | NONE | Bfp, eagg | A | None | None | |
| 25C | 0-1 years | Male | ESBL,AMPC | Imp, vim | NA-TZP | Bfp, eagg | B1, D | None | None | |
| 26C | 0-1 years | Male | AMPC | Ndm, vim, dha, cmy, ctx | CTX-NA | Eae | NONE | sul1 | None | |
| 27C | 3-5 years | Male | ESBL | Tem, shv | NF | Eae, eaf, eagg | NONE | sul1 | None | |
| 28C | 0-1 years | Male | NONE | None | NONE | Eagg | B2 | None | None | |
| 29C | 3-5 years | Male | AmpC | Act, dha | NF | Eagg | NONE | sul1 | None | |
| 30C | 0-1 years | Male | ESBL | Tem | NA | None | B2 | None | None | |
| 31C | 3-5 years | Female | ESBL | Tem | NONE | None | NONE | None , | None | |
| 32C | 0-1 years | Male | ESBL | Tem, shv | CTX | Eaf, eagg | NONE | tetA, tetC | None | |

| | | | | | | | | | | |
|---|---|---|---|---|---|---|---|---|---|---|
| 33C | 3-5 years | Female | AMPC,AmpC | Imp, vim | NF-CTX | eae | B1, F | tetA, sul1, tetB | None | |
| 34C | 1- 3 years | Female | ESBL,AMPC | Ndm, imp, act | NONE | Eaf, eagg, hyla | NONE | None | None | |
| 35C | 1- 3 years | Male | AMPC | Imp | NA | Bfp, eaf | NONE | sul1 | GyrA | 83 |
| 36C | 0-1 years | Female | ESBL | Tem, oxa | NONE | est | D | None | None | |
| 37C | 1- 3 years | Female | NONE | Ctx | NA | Eae, eaf | A | sul1 | None | |
| 38C | 1- 3 years | Male | AmpC | Dha, cmy | NA | None | B2 | sul1 | None | |
| 39C | 0-1 years | Male | ESBL | Tem, shv | NF | Bfp, eae, eagg | NONE | sul1 | None | |
| 40C | 0-1 years | Male | ESBL | Tem, ctx, oxa | NF | bfp | B2 | sul1 | None | |

Note: Each group included 40 subjects and were categorized as diarrhoeal (O), non-diarrhoeal (I) and healthy (C).
