# Supplementary material for: Changing paradigm of antibiotic resistance amongst Escherichia coli isolates in Indian pediatric population
Source: PLoS One. 2019 Apr 17;14(4):e0213850. doi: 10.1371/journal.pone.0213850 (PMC6469777; doi:10.1371/journal.pone.0213850)
Supplement: S2 Table — (PDF) [file pone.0213850.s003.pdf]

S2 Table: Frequency of resistance to antimicrobial agents of *E. coli* isolates from the three study groups.

Antibiotics	Group 1 n=40 (%)	Group 2 n=40 (%)	Group 3 n=40 (%)	Total n =120 (%)	P value
Norfloxacin(10µg)	9(22.5)	7(17.5)	10(25)	26(21.66)	0.709
Cefotaxime (30µg)	27(67.5)	33(82.5)	7((17.5)	67(55.83)	0.00*
Imipenem (10µg)	12(32.5)	5(12.5)	1(2.5)	18 (15)	0.002*
Meropenem (10µg)	2(5)	1(2.5)	0	*3(2.5)*	0.358
Ceftazidime(30µg)	8(20)	4(10)	0	12(10)	0.011*
Azetronam (30µg)	5(12.5)	6(15)	0	11(9.1)	0.044*
Nalidixic acid(30µg)	8(20)	0	0	14(11.66)	0.00*
Amoxicillin/clavulanic acid(20/10µg)	1(2.5)	2(5)	0	3(2.5)	0.358
Gentamicin (10µg)	15(37.5)	14(35)	2(5)	31(25.83)	0.01*
Ciprofloxacin (5µg)	7(17.5)	4(10)	1(2.5)	12(10)	0.082
Ampicillin(10µg)	17 (42.5)	9 (22.5)	4 (10)	30 (25)	0.01*
Amikacin (30µg)	9(22.5)	14(35)	0	23(19.1)	0.00*
Polymyxin B (300µg)	1(2.5)	0	0	1(0.83)	0.364
Cefotaxime + clavulanic acid (30/10µg)	0	1(2.5)	0	1(0.83)	0.364
Ceftriaxone (30µg)	0	1(2.5)	1(2.5)	2(1.66)	0.601
Piperacillin+tazobactam (100/10µg)	10(25)	9(22.5)	2(5)	21(17.5)	0.037*

***Statistically significant; Antibiotic frequencies are presented as absolute numbers (n) with percentage in parentheses; Group 1 - diarrhoeal, Group 2 - non-diarrhoeal, and Group 3 - healthy.**
